# Microbes and masculinity: Does exposure to pathogenic cues alter women’s preferences for male facial masculinity and beardedness?

**DOI:** 10.1371/journal.pone.0178206

**Published:** 2017-06-08

**Authors:** Toneya L. McIntosh, Anthony J. Lee, Morgan J. Sidari, Rebecca E. Stower, James M. Sherlock, Barnaby J. W. Dixson

**Affiliations:** 1 School of Psychology, University of Queensland, Brisbane, Queensland, Australia; 2 Institute of Neuroscience and Psychology, University of Glasgow, Scotland, United Kingdom; Institute of Tropical Medicine, JAPAN

## Abstract

Women’s preferences for men’s androgen dependent secondary sexual traits are proposed to be phenotypically plastic in response to exposure to pathogens and pathogen disgust. While previous studies report that masculinity in facial shape is more attractive to women who have recently been exposed to pathogenic cues and who are high in self-reported pathogen disgust, facial hair may reduce male attractiveness under conditions of high pathogens as beards are a possible breeding ground for disease carrying ectoparasites. In the present study, we test whether women’s preferences for beardedness and facial masculinity vary due to exposure to different pathogenic cues. Participants (N = 688, mean age + 1SD = 31.94 years, SD = 6.69, range = 18–67) rated the attractiveness of facial composite stimuli of men when they were clean-shaven or fully bearded. These stimuli were also manipulated in order to vary sexual dimorphism by ±50%. Ratings were conducted before and after exposure to one of four experimental treatments in which participants were primed to either high pathogens (e.g. infected cuts), ectoparasites (e.g. body lice), a mixture of pathogens and ectoparasites, or a control condition (e.g. innocuous liquids). Participants then completed the three-domain disgust scale measuring attitudes to moral, sexual and pathogen disgust. We predicted that women would prefer facial masculinity following exposure to pathogenic cues, but would show reduced preferences for facial hair following exposure to ectoparasites. Women preferred full beards over clean-shaven faces and masculinised over feminised faces. However, none of the experimental treatments influenced the direction of preferences for facial masculinity or beardedness. We also found no association between women’s self-reported pathogen disgust and their preferences for facial masculinity. However, there was a weak positive association between moral disgust scores and preferences for facial masculinity, which might reflect conservatism and preferences for gender typicality in faces. Women’s preferences for beards were positively associated with their pathogen disgust, which runs contrary to our predictions and may reflect preferences for high quality individuals who can withstand any costs of beardedness, although further replications are necessary before firm conclusions can be made. We conclude that there is little support for pathogenic exposure being a mechanism that underpins women’s directional preferences for masculine traits.

## Introduction

Ecological conditions influence female capacity and motivation to choose mates, dramatically altering how sexual selection shapes the evolution of attractive traits [1,2]. Where condition dependent traits are concerned, parasitic infection may reduce an individual’s ability to signal their quality via ornamentation [3] and sexual selection via mate choice for condition-dependent ornaments may be stronger under prevailing conditions of high pathogen richness [4]. While a fitness advantage to females who select males with stronger past immunity is plausible, effect sizes are likely small in comparison to the advantages gained by selecting a male in good current condition [5]. This plasticity in mate preferences may contribute to the maintenance of variation in sexually attractive ornaments.

Adaptation to prevailing environmental conditions, particularly pathogens and disease carrying parasites, has shaped the evolution of human biological immune systems [6]. Human cognition may also have been shaped by natural selection to identify and avoid pathogenic stimuli [7]. This *behavioural immune system* has been implicated in a host of human interpersonal behaviors [7,8], including mate preferences for individuals advertising disease resistance or genotypes that confer immunity from infection [9,10]. For example, sexual dimorphism in craniofacial morphology, which includes the brow ridge, jaw, byzygomatic width and facial length and is collectively termed facial masculinity [11], may be associated with long-term health [12], disease resistance [13] and some aspects of immune response [14]. Thus, facial masculinity may reliably indicate current condition and enhance attractiveness to women.

While facial masculinity may communicate aspects of mate quality, when energetic resources are prioritised towards mating effort health and paternal investment may become compromised [15,16]. More facially masculine men report less interest in long-term relationships, higher infidelity [17,18] and are judged as looking less caring and paternally investing [19,20]. Women may bypass any possible costs of selecting a less prosocial and paternally investing partner under conditions wherein the benefits of greater masculinity are particularly high [21]. Thus, preferences for facial masculinity are stronger under ecological conditions where survival is compromised [22–25]. Specifically, women’s preferences for facial masculinity in men are also positively associated with individual differences in self-reported pathogen disgust [26,27] and are stronger immediately following exposure to pathogenic stimuli [28]. Taken together, these findings suggest that women’s preferences for facial masculinity vary in response to perceived pathogen threat. However, we note that research in this area is mixed, with some studies finding no association between women’s facial masculinity preferences and pathogen threat [29], or providing contradictory evidence indicating that the relationship is more complex [30–32].

Natural selection is also strongly implicated in reducing overall body hair in humans to maintain thermal homeostasis during upright stance and bipedal locomotion [33] and reduce the burden of disease carrying ectoparasite [34–36]. However, humans have retained conspicuous patches of hair on the head and body, which is highly sexually dimorphic in the case of beardedness, chest and trunk hair [37,38]. Body hair may provide the ideal conditions for ectoparasites to proliferate and transmit diseases [36]. Vectors that transmit disease via cutaneous contact, such as insect bites, elicit disgust responses that are distinct from other dimensions of pathogen disgust [39]. Insects and invertebrates are rated as highly disgusting [40], elicit strong disgust responses [41] and cause participants to report increased grooming behaviors when the threat of disease is more salient [42]. According to the ectoparasite avoidance hypothesis, reduced hirsutism was further elaborated upon via sexual selection [36, 43]. Indeed, in only a minority of cultures, such as the UK and Cameroon, do women prefer male chest hair [44,45], whereas hairless chests are most attractive in Brazil, the USA, the Czech Republic, China, New Zealand, Finland, Turkey, and Slovakia [46–49]. Beards may harbour bacteria or parasites [50] and women rated bearded faces as dirtier than clean-shaven faces [51], which may contribute to the variation in women’s preferences for men’s facial hair (for reviews see [49, 52]). However, whether women’s preferences for men’s beards vary following exposure to ectoparasites or differ due to individual differences in pathogen disgust remains to be determined.

The present study tested whether variation in women’s preferences for facial masculinity and beardedness were phenotypically plastic in response to exposure to pathogens and individual differences in pathogenic disgust. Participants rated the attractiveness of male faces varying in masculinity (±50% masculinity) and beardedness (clean-shaven and fully bearded) before and after exposure to pathogenic stimuli. Participants were assigned to one of four treatments in which they either saw images of ectoparasites (e.g. a burrowing tick), pathogens (e.g. open infected cuts), a combination of ectoparasites and pathogens (a mixed treatment) or neutral stimuli (a control treatment). We hypothesised that women would assign higher attractiveness ratings to masculine faces after exposure to the pathogen treatment than the control treatment [28]. We also hypothesised that women would rate fully bearded men as less attractive than clean-shaven men following exposure to the ectoparasite treatment than those assigned to the control treatment [36,43]. We included a mixed treatment comprised of both ectoparasites and pathogenic stimuli as both types of stimuli can occur simultaneously, but insects and other ectoparasites activate distinct disgust responses compared to pathogenic stimuli [39]. We speculated that if preferences for masculinity and clean-shaven faces were stronger following exposure to pathogens and ectoparasites respectively, this effect would be more pronounced than any preferences activated by the mixed treatment. We also predicted based on past research that women’s preferences for facial masculinity would be positively correlated with their self-reported pathogen disgust [26,27], while preferences for facial hair would be negatively correlated with pathogen disgust [53]. In testing these predictions, we aimed to expose whether specific context dependent preference functions underpin the attractiveness of facial masculinity and beardedness to women.

## Material and methods

### Ethics statement

The current study was approved by the human ethics committee at the University of Queensland (approval # 16-PSYCH-4-58-TS).

### Facial stimuli

#### Facial hair photographs

Thirty-seven men (mean age ± SD = 27.86 ± 5.75 years) of European ethnicity were photographed posing neutral facial expressions in front and profile view using a Canon digital camera (8.0 megapixels resolution) positioned 150 cm from the participant under controlled lighting. Males were photographed when clean-shaven and with 4–8 weeks of natural beard growth [54].

#### Facial composites

The clean-shaven and fully bearded versions of the male photographs were used to construct composite stimuli using the Webmorph software package [55]. Composite images were created by randomly selecting five of the thirty-seven individuals and averaging both the clean-shaven images and the corresponding bearded versions of the same individuals. This was done on the basis of 189 landmarks on the face [54].

#### Facial masculinity manipulation

**Fig 1:**
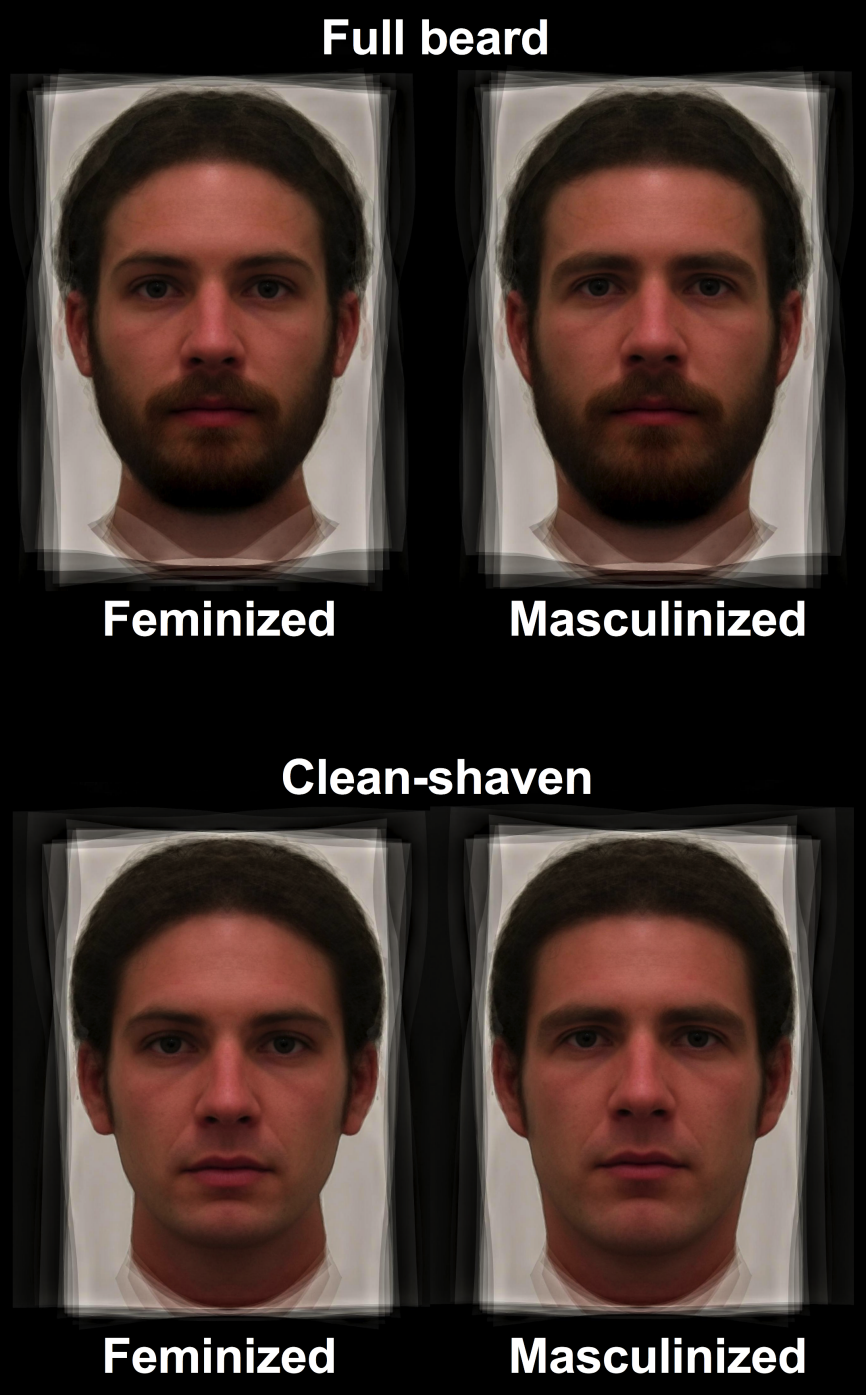
Examples of the male stimuli used in this study. Images depict composites of the same five individuals with full beards (upper images) and when clean-shaven (lower images), as well as when manipulated to appear 50% more masculinized (right images) or 50% less masculinized (i.e. feminized; left images). These images are computer-generated and do not represent the true identities of any individual.

A composite male and female face were created from a separate face set of 40 male and 40 European females based on the same 189 landmarks. To manipulate facial masculinity, the linear shape differences between the average male and female faces were applied to the clean-shaven and bearded composites at ±50% while keeping colour and textural information of the original face constant. This, effectively, manipulated these images on the dimension representing sexual dimorphism while retaining the identity of the original composite [Fig 1]. This method is standard for manipulating sexual dimorphism in facial images [19, 56].

### Stimuli for priming pathogen exposure

#### Cues to pathogens and control images

Images were sourced from Curtis et al [57], which quantified disgust ratings in a large sample of men and women for pairs of stimuli varying in apparent high or low pathogen contagion. For example, one stimulus pairing depicted a bowl filled with liquid resembling bile (i.e. high pathogen) and a corresponding image depicting a bowl filled with an innocuous blue liquid (i.e. low pathogens). Of the seven pairs of stimuli, six pairs were consistently judged as representing both high and low pathogenic stimuli [57]. For the current study, these images were sourced from a high-resolution PDF and were used in the high pathogen, mixed and control experimental treatments. These stimuli have also been used in past studies to uncover whether exposure to cues of high pathogens enhance preferences for facial shape in women and men [28].

#### Ectoparasites

Eight images of ectoparasites were sourced from the Internet using a Google image search. These images included various species of burrowing ticks, such as the sheep tick (*Ixodes ricinus*) and the Australian paralysis tick (*Ixodes holocyclus*), as well as the body louse (*Pediculus humanus humanus*) and the pubic louse (*Pthirus pubis*). As these stimuli are of ectoparasites it was appropriate to present them as they occur on the skin, either attached to hair, burrowing into the skin or attached to the surface of the skin. To validate whether these stimuli were effective in eliciting disgust, we recruited 99 female participants online to rate the ectoparasite stimuli and the control stimuli from Curtis et al [57] using the same scale (1 = not disgusting at all to 5 = extremely disgusting). Ratings showed good internal consistency for both the ectoparasite (Cronbach’s alpha = 0.91) and control (Cronbach’s alpha = 0.76) images. Disgust ratings were significantly higher for the ectoparasite stimuli (Mean = 3.65, S.D = 0.90) than the control (1.33, S.D = 0.42) images (*t*_98_ = 26.11, *p* < 0.001) and the effect size was large (*d* = 3.31).

#### Experimental treatments used to manipulate pathogen exposure

The pathogens and the ectoparasite stimuli were used to populate four experimental treatments to test our hypotheses regarding how exposure to pathogenic cues influence women’s directional preferences for men’s secondary sexual traits. The first treatment aimed to prime participants to high pathogens and presented only the images that represented high pathogens [28, 57], this treatment is hereafter referred to as the ‘pathogens treatment’. The second treatment aimed to prime participants to high ectoparasite loads and used only the ectoparasite stimuli and is hereafter referred to as the ‘ectoparasite treatment’. We created a treatment in which an even number of stimuli used in the pathogen and ectoparasite treatments were combined, which is hereafter referred to as the ‘mixed treatment’. Finally, we included a control treatment in which only the non-pathogenic stimuli from Curtis et al [57] were presented.

#### Demographics

Participants reported their sexuality using the seven-point Kinsey sexual orientation scale where 0 = exclusively heterosexual and 6 = exclusively homosexual [58]. They then provided their age (in years), biological sex (male, female, other), ethnicity (open question) and relationship status (single or currently in a relationship).

#### Three-domain disgust scale

All participants completed the three domains of disgust scale, which measures attitudes to performing or observing 21 different actions using a 7-point Likert scale where 0 = not at all disgusting, 6 = extremely disgusting [59]. The 21 attitudes comprise three separate but interrelated domains of disgust: moral disgust (e.g., forging someone’s signature on a legal document), sexual disgust (e.g., performing oral sex), and pathogen disgust (e.g., accidentally touching a person’s bloody cut).

#### Procedure

The current study was administered online. Past research has shown that online experiments, including those using priming procedures, produce comparable results to studies undertaken in laboratory settings [60,61]. Upon entering the online experiment, participants first read an information sheet and provided consent to participate in the study. They were informed they would see a series of 20 faces and were asked to look carefully at each image and rate their sexual attractiveness on a scale of 0 (extremely unattractive) to 100 (extremely attractive). The 20 faces depicted five composites of the same individuals when bearded and clean-shaven that had been manipulated to appear 50% more masculine and 50% less masculine (Fig 1). Each participant rated the stimuli in a randomized order.

Participants began by rating the 20 faces for attractiveness prior to receiving the priming condition (i.e. a pre-rating block). Participants were then randomly assigned to one of the four priming treatments (pathogen, ectoparasite, mixed or control) during which they saw a series of seven images presented in a randomized order for three seconds. The exposure to the seven stimuli was repeated a total of three times [28]. After the priming condition, participants rated the 20 faces again for attractiveness (i.e. a post-rating block). Participants were then asked to complete the Kinsey scale for sexual orientation, answer the demographic questions, and complete the three domains of disgust scale.

#### Participants

Participants completed the experiment online via Amazon Mechanical Turk (M-Turk) in return for $1.50. M-Turk is an online marketplace that employs research participants via crowdsourcing and has been particularly useful for behavioural researchers seeking non-student samples [62]. An initial screening survey was posted on MTurk in which demographic data were collected. Participation was conditional on being between the ages of 18–44, heterosexual, female and residing in an English speaking country (Australia, Canada, New Zealand, UK, USA). To minimize any biases in completing the experiment, participants were informed that they would judge faces for attractiveness without specifying facial masculinity or beardedness. A total of 802 participants (mean age + 1SD = 31.84, 6.73) were eligible. We also asked participants if they had been honest regarding their stated gender while assuring them that they would not be penalized and would still receive their payment for participating. Thirty-two males were removed, along with 82 additional female participants who did not complete the survey, leaving a final sample of 688 females (31.94 years, SD = 6.69). Five hundred and thirty-nine (78%) of participants were in a relationship (32.22 years, SD = 6.53) while 149 (22.2%) were not in relationships (30.93 years, SD = 7.12). Participants were predominantly heterosexual (95.9%), while 2.2% were heterosexual but more than incidentally homosexual, 0.9% were bisexual, 0.1% homosexual and 0.9% were asexual. Ethnicities were as follows: 78.6% were Caucasian, 8.4% African-America, 7.4% were Asian, 0.6% were Native American, 0.1% were native Pacific islander and 4.8% identified as other.

The 688 participants were evenly spread among the four experimental treatments, so that the ectoparasite treatment had 175 participants (age = 31.40 years, SD = 6.55), the pathogens treatment had 172 participants (age = 32.09 years, SD = 6.58), the mixed treatment had 177 participants (age = 32.05 years, SD = 6.15), and the control group had 164 participants (age = 32.24 years, SD = 7.46). The ages of participants were not significantly different between treatments (F_3,687_ = 0.54, P = 0.659) and ethnicity and sexual orientation was comparable across the experimental treatments (S1 Table).

#### Statistical analysis

In *Analysis 1*, we report whether pathogen priming caused directional shifts in preferences. Attractiveness ratings for the stimulus images within each category of facial hair (clean-shaven, bearded) and facial masculinity (high, low) showed strong internal consistency (all Cronbach alphas ≥ 0.90; S2 Table). Thus, we averaged attractiveness ratings across the five stimuli within each of the four facial categories (i.e. full beard high masculinity; full beard low masculinity; clean shaven high masculinity; clean shaven low masculinity). These ratings were dependent variables in a repeated measures ANOVA where facial masculinity (high masculinity; low masculinity), beardedness (full beard; clean shaven) and time (pre-treatment; post-treatment) were within subject factors and experimental treatment (ectoparasites; pathogen; mixed; control) was the between-subjects factor.

In *Analysis 2*, we report whether individual differences in disgust are associated with preference for beardedness and facial masculinity. Each participant rated 40 faces (both pre- and post-manipulation), resulting in 27480 observations. These data are hierarchical in nature, as each of the attractiveness ratings (Level 1) are nested within the participant who made them (Level 2). As such, we analysed the data using mixed effects modelling (for an explanation of this technique and its advantages over other approaches, see [63]). On Level 1, participants’ preference for each trait is revealed by the association between characteristics of the face (either masculinised or feminised, or clean-shaven or bearded) and the outcome attractiveness rating. We tested the influence of the Level 2 predictors on these associations. Moral and sexual disgust were also included to ensure any effect of pathogen disgust did not simply reflect an effect of general disgust. These scales showed good internal reliability (Cronbach alphas = 0.93, 0.82 and 0.83 respectively). All predictors were entered simultaneously. To facilitate interpretation, all continuous predictors were standardised, while dichotomous variables were effect coded (-.5 or .5). This analysis has previously been used to examine the influence of pathogen disgust and facial preferences [29]. Even though stimuli identity was repeated within participants, we do not expect this would influence results given the high homogeneity of stimuli. Regardless, we ran a model including a random effect of stimuli identity and, as expected, this model failed to converge. Below, we report analyses using both pre- and post-manipulation attractiveness rating as the outcome variable. We also ran the analyses using only the pre-manipulation ratings, which did not change the pattern of results for hypothesised effects (S3 Table and S4 Table).

## Results

### Analysis 1: Does pathogen priming cause directional shifts in masculinity preferences?

There were significant main effects of facial hair and facial masculinity on attractiveness ratings (Table 1). Beards received higher ratings of attractiveness than clean-shaven faces (*t*_687_ = 18.48, *P* < 0.001) and high masculinity received higher ratings of attractiveness than low masculinity (*t*_687_ = 10.84, *P* < 0.001).

**Table 1:** Repeated-measures ANOVA testing the effect of beardedness (clean-shaven, full beard), masculinity (+50%, -50%), time (pre, post) and pathogen treatment (ectoparasites, pathogens, mixed, and control) on women’s attractiveness ratings of male faces.

	d.f._n_	d.f._d_	*F*	*P*	*η*_*p*_^*2*^
Facial hair	1	684	340.41	<0.001	0.332
Facial masculinity	1	684	117.86	<0.001	0.147
Time	1	684	0.83	0.362	0.001
Treatment	3	684	2.03	0.109	0.009
Facial hair x facial masculinity	1	684	18.91	<0.001	0.027
Facial hair x treatment	3	684	0.28	0.843	0.001
Facial hair x time	1	684	26.29	<0.001	0.037
Facial masculinity x treatment	3	684	1.15	0.327	0.005
Facial masculinity x time	1	684	0.10	0.754	<0.001
Time x treatment	3	684	2.10	0.099	0.009
Facial hair x facial masculinity x treatment	3	684	0.28	0.838	0.001
Facial hair x facial masculinity x time	1	684	0.05	0.817	<0.001
Facial hair x time x treatment	3	684	1.20	0.310	0.005
Facial masculinity x time x treatment	3	684	0.19	0.905	0.001
Facial hair x facial masculinity x time x treatment	3	684	0.67	0.571	0.003

**Fig 2:**
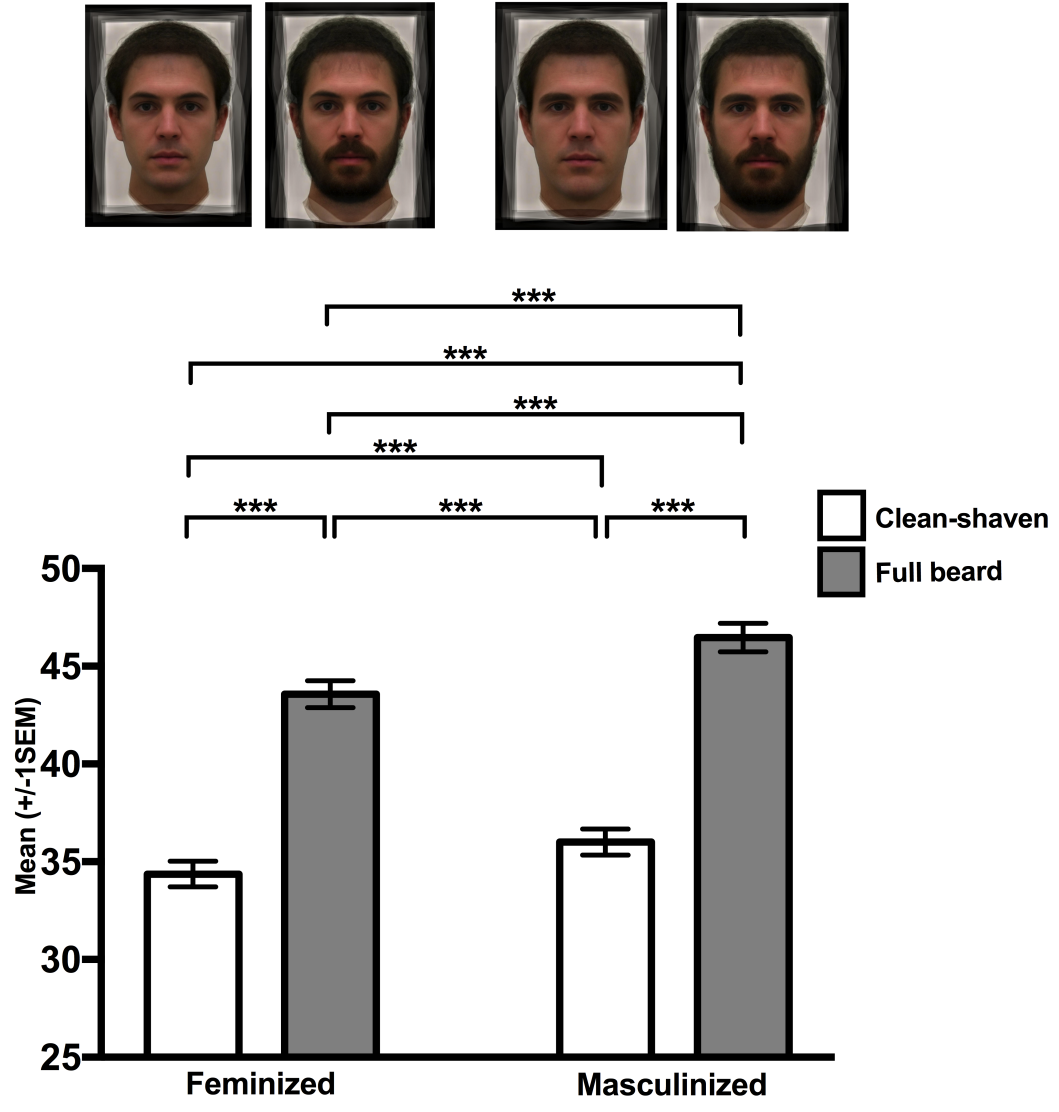
The role of facial masculinity and beardedness in women’s judgments of men’s attractiveness. Data are the mean attractiveness ratings (± 1 SEM) of feminized and masculinized face, split by clean-shaven (open bars) and fully bearded faces (grey bars). *** = p < 0.001. The images at the top of the figure are computer-generated and do not represent the true identities of any individual.

The significant facial hair × masculinity interaction (Table 1), shows women's ratings of attractiveness were higher for high masculinity compared to low masculinity within clean-shaven (*t*_687_ = 10.31, *P* < 0.001) and bearded (*t*_687_ = 7.34, *P* < 0.001) conditions and this effect was more pronounced in bearded (*d* = 0.16) than clean-shaven conditions (*d* = 0.09). Bearded faces with high and low masculinity were more rated as more attractive than clean-shaven faces with high and low masculinity (all *t*_687_ ≥ 14.30, *P* < 0.001; Fig 2).

**Fig 3:**
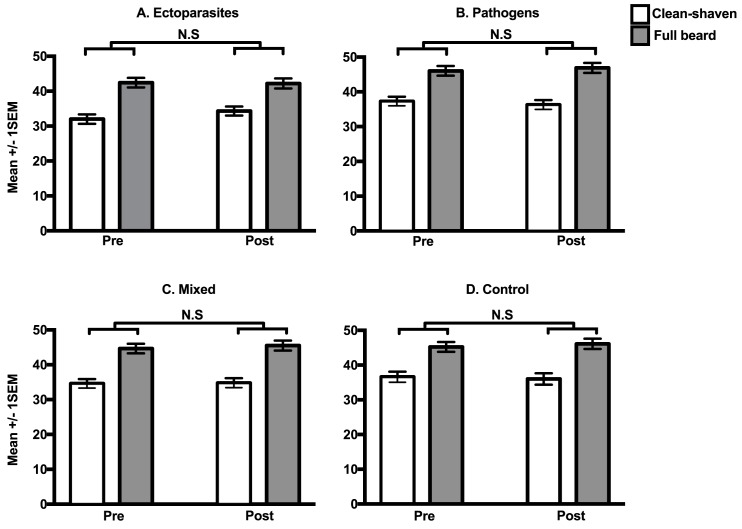
The effect of treatment on women’s preferences for men’s beardedness. Data are the mean attractiveness ratings (± 1 SEM) of clean-shaven (open bars) and fully bearded faces (grey bars) pre and post exposure to ectoparasites (**A.**), pathogens (**B.**), mixed (**C.**) and control (**D.**) treatments. N.S = Not statistically significant.

**Fig 4:**
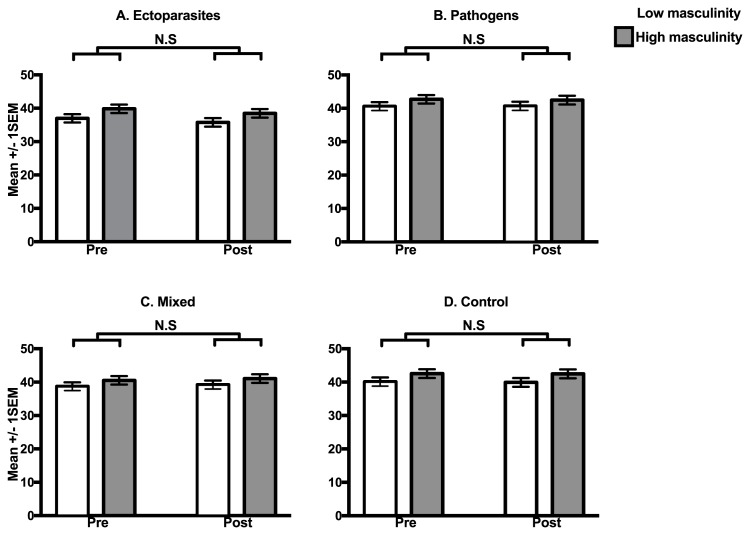
The effect of treatment on women’s preferences for men’s facial masculinity. Data are the mean attractiveness ratings (± 1 SEM) of feminised (open bars) and masculinised faces (grey bars) for judgments pre and post exposure to ectoparasites (**A.**), pathogens (**B.**), mixed (**C.**) and control (**D.**) treatments. N.S = Not statistically significant.

There was a significant main effect of time (Table 1), such that ratings given prior to the priming treatments were slightly higher than ratings after the priming treatments. There was also a significant facial hair × time interaction on ratings of attractiveness (Table 1), which reflects that women's ratings of attractiveness were significantly lower for clean-shaven faces post-treatment than pre-treatment (*t*_687_ = 3.31, *P* < 0.001) but not bearded faces (*t*_687_ = 1.60, *P* = 0.110). However, there were no statistically significant interactions involving pathogen treatment (Table 1), for either beardedness (Fig 3) or facial masculinity (Fig 4). This finding remained when using a differential between post-treatment and pre-treatment attractiveness ratings as the dependent variable (S5 Table).

We ran additional analyses to test whether participant’s relationship status influenced any interactions of pathogen treatment on preferences. There were no statistically significant interactions involving current relationship status and pathogen treatment, for beardedness or facial masculinity (S6 Table). There was a significant facial masculinity × relationship status interaction on ratings of attractiveness (S6 Table). This reflects that while women's ratings of attractiveness were significantly higher for high masculinity over low masculinity, this effect was larger among women currently in relationships (*t*_538_ = 11.19, *P* < 0.001; *d* = 0.16) than women not in relationships (*t*_148_ = 2.25, *P* = 0.026; *d* = 0.06). Comparisons between participants currently in a relationship and those who were not revealed no significant differences in preferences for high facial masculinity (*t*_686_ = 1.24, *P* = 0.215) or low facial masculinity (*t*_686_ = 0.19, *P* = 0.852). All other interactions involving relationship status were not statistically significant (S6 Table).

### Analysis 2: Are individual differences in disgust associated with masculinity preferences?

An empty model with no predictors found that the intraclass correlation (i.e., the proportion of total variance that is between-individuals) indicated that variance in participants’ attractiveness ratings existed at both levels (ICC = .50), confirming the use of mixed effects modelling. The variance components are reported in the Supplementary Materials (S7 Table).

The fixed effects coefficients are reported in Table 2.

**Table 2:** The fixed effects coefficients (and standard errors) and associated 95% confidence intervals for the influence of moral disgust, sexual disgust, pathogen disgust, as well as sexual dimorphism and beardedness on attractiveness ratings.

	γ (SE)	95% CI
Intercept	34.22 (.61)	33.02, 35.43*
Moral Disgust	1.13 (.68)	-.20, 2.46
Sexual Disgust	-.19 (.71)	-1.59, 1.22
Pathogen Disgust	-2.69 (.73)	-4.17, -1.23*
Preference for Beardedness	9.86 (.18)	9.50, 10.21*
Moral Disgust	-.18 (.19)	-.56, .20
Sexual Disgust	-1.63 (.21)	-2.04, -1.23*
Pathogen Disgust	.79 (.21)	.38, 1.20*
Preference for masculinity	2.27 (.18)	1.92, 2.63*
Moral Disgust	.45 (.19)	.07, .83*
Sexual Disgust	-.04 (.21)	-.45, .36
Pathogen Disgust	-.29 (.21)	-.70, .12

* 95% confidence interval does not contain 0, indicating statistical significance.

**Fig 5:**
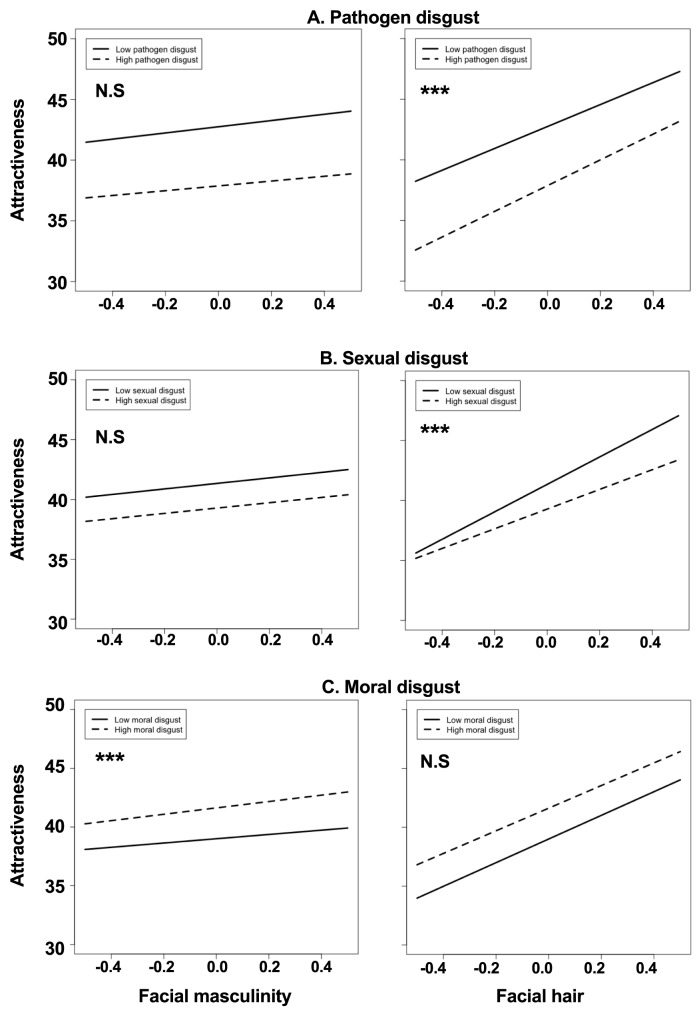
The associations between the three domains of disgust and women’s preferences for men’s facial masculinity and beardedness. Data show the effects of low (-1SD) and high (+1SD) pathogen (A.), sexual (B.), and moral (C.) disgust sensitivity on women’s preference for facial masculinity (left) and facial masculinity (right). *** = 95% confidence interval does not contain 0, indicating statistical significance. N.S = Not statistically significant.

Overall, bearded faces were rated as more attractive compared to their clean-shaven counterparts. Pathogen disgust significantly moderated this relationship, such that as pathogen disgust increased, preference for bearded faces also increased (Fig 5A). However, we also found that sexual disgust significantly negatively impacted on preferences for bearded faces (Fig 5B). Overall, masculinised faces were rated as more attractive than the feminised versions. Only moral disgust moderated this relationship, such that preference for masculinised faces increased as moral disgust increased (Fig 5C). There was also a main effect of pathogen disgust on attractiveness ratings, such that participants with higher pathogen disgust overall gave lower attractiveness ratings.

## Discussion

We tested whether women adjust their preferences for facial masculinity and beardedness following exposure to pathogenic stimuli. Past studies reported that women’s preferences for sexual dimorphism in male faces increase following exposure to visual cues of high pathogens [28] and vignettes describing high pathogens [64]. However, we did not find that women’s preferences for more masculine faces were greater following exposure to pathogenic stimuli compared to pathogen irrelevant stimuli. To manipulate pathogenic cues, we used stimuli that had been previously shown to elicit strong disgust responses [57] and that had been found to activate women’s preferences for sexual dimorphism in male faces [28]. Our findings join a growing literature that questions whether exposure to threat of disease influences women’s preferences for facial masculinity [31,32].

We also tested whether the ectoparasite avoidance hypothesis, wherein women’s preferences for men’s facial and body hair should decrease following exposure to ectoparasites [36], explained reduced preferences for men’s beardedness. We primed participants using images depicting various species of lice and ticks on or burrowing into the skin and attached to body hair, which were judged as significantly more disgusting compared to control images. However, we did not find women’s preferences for beards were reduced following exposure to ectoparasites, or any of the other pathogenic conditions. Previous research has shown that facial hair enhances perceptions of angry facial expressions but reduces the impact of smiles [65]. Future research employing more dynamic stimuli than the highly controlled composite stimuli with neutral facial expressions might be beneficial for testing effects of avoidance of facial hair following exposure to pathogenic stimuli. Our findings from the present study support past research showing that priming to ectoparasites and pathogens do not alter women’s preferences for men’s chest and trunk hair [66] and suggests that the ectoparasite avoidance hypothesis may not explain variation in women’s preference for androgen dependent facial or body hair in men.

In addition to testing whether priming to pathogenic stimuli has causal directional effects on women’s preferences for beards and facial masculinity, we also tested whether individual differences in self-reported pathogen disgust sensitivity were associated with masculinity and beardedness preferences. For women’s preference for facial masculinity, we found no association with their self-reported pathogen disgust. This contradicts previous research reporting that women’s preferences for facial masculinity were positively associated with their pathogen disgust [26,27], but supports the growing body of research that has failed to find this association [29]. A potential reason for our failure to find any association between facial masculinity preferences and self-reported pathogen disgust may be due, in part, to our use of a continuous rating scale, which has been found to be less powerful compared to forced-choice paradigms [30]. We did find a weak, positive association between moral disgust scores and preferences for facial masculinity, which may reflect conservatism and preferences for gender typicality in faces [67]; although, previous research has failed to find an effect of moral disgust sensitivity on facial masculinity preferences [27,29]. Combined with other recent findings [29,68], our results further question whether an association exists between pathogen concerns and preference for facial masculinity.

We also found that women’s preferences for men’s beardedness were positively associated with self-reported pathogen disgust, but negatively associated with sexual disgust. Following the ectoparasite avoidance hypothesis [53], we had predicted that women’s pathogen disgust would be negatively associated with preferences for facial hair. The positive association we report here could be interpreted as support for parasite resistance handicap hypotheses [69], as facial hair has the potential to harbor disease carrying ectoparasites, that may have impact on male survivability ancestrally [34–36], so that only high quality males could maintain beards. However, evidence from medical studies that beards harbor bacteria is mixed [50,70,71] and we found no causative effect of priming pathogens on preferences for beards. The negative association between preferences for beards and self-reported sexual disgust might reflect preferences for cues of masculine conservatism, as facial hair is associated with masculinity and dominance [72–76], political conservatism [77] and the endorsement of gender typical roles in heterosexual relationship [78,79]. However, men’s grooming habits vary markedly between and within populations [80,81] and associations between the choice to have facial hair across cultures is not clearly associated with demographic factors linked to economics and gender inequality [81]. Thus, we treat the subtle associations we found here between self-reported disgust and preferences for facial masculinity and beardedness with caution and suggest further replication from additional study populations is required.

The intersection between biology and culture in shaping mate preferences has some potential to uncover new mechanisms underpinning the maintenance of variation in preferences for attractive traits [82]. Variation within and between cultures in how female choice may shape men’s grooming habits has only recently begun to receive attention [81,83] and has not implicated a role for pathogen richness in maintaining cross-cultural variation in facial hair grooming or women’s facial hair preferences [81]. Further, while cross-cultural studies have reported that women’s preferences for facial masculinity were strongest in countries with higher disease burdens and reduced life expectancy [22–24], studies that included people from outside of so-called WEIRD (Western, Educated, Industrialized, Rich and Democratic) societies [84] did not find this effect [31,85]. Further, studies implicating individual differences in women’s fertility have also not found that preferences for beardedness or body hair are greater when fertility is higher [48, 86–88]. Finally, among identical and non-identical twins, 38% of the variance in women’s facial masculinity preferences were due to genetic variation, while self-reported sociosexuality, fertility and pathogen disgust accounted for less than 1% [32]. Taken together, the results from the current and other recent studies call into question whether facets of the behavioral immune system explain variation in women’s preferences for men’s masculine facial traits.

## Supporting information

S1 File This file contains the data reported in this manuscript. (SAV)

S1 Table Participants sexual orientation and ethnicity split by experimental treatment. (DOCX)

S2 Table Cronbach’s alphas representing the inter-rater reliability for the 5 stimulus images within each stimulus category for ratings of attractiveness ratings. (DOCX)

S3 Table The variance components (random effects) for the models predicting attractiveness ratings when only including pre-manipulation trials. (DOCX)

S4 Table The fixed effects coefficients (and standard errors) and associated *95*% confidence intervals for the influence of moral disgust, sexual disgust, pathogen disgust, as well as sexual dimorphism and beardedness on attractiveness ratings only including pre-manipulation trials. (DOCX)

S5 Table Repeated-measures ANOVA testing the effect of beardedness (clean-shaven, full beard), masculinity (+50%, -50%) and pathogen treatment (ectoparasites, pathogens, mixed, and control) on the differential between women’s post-treatment and pre-treatment attractiveness ratings of male faces. (DOCX)

S6 Table Repeated-measures ANOVA, with the beard (clean-shaven, full beard), masculinity (+50%, -50%) and time (pre, post) as within-subjects factors and pathogen treatment (ectoparasites, pathogens, mixed, and control) and relationship status (in a relationship, single) as between-subjects factors. (DOCX)

S7 Table The variance components (random effects) for the models predicting attractiveness ratings for Analysis 2. (DOCX)
