# Supplementary material for: Microbes and masculinity: Does exposure to pathogenic cues alter women’s preferences for male facial masculinity and beardedness?
Source: PLoS One. 2017 Jun 8;12(6):e0178206. doi: 10.1371/journal.pone.0178206 (PMC5464545; doi:10.1371/journal.pone.0178206)
Supplement: S1 Table — (DOCX) [file pone.0178206.s002.docx]

| **Table S1**. Participants sexual orientation and ethnicity split by experimental treatment. | | | | | | | | |
| --- | --- | --- | --- | --- | --- | --- | --- | --- |
| **Sexual orientation*** | | | | | | | | |
| **Treatment** | **1** | **2** | **3** | **4** | **5** | **6** | **7** | **8** |
| Pathogens | 142 (80.2%) | 28 (15.8%) | 4 (2.3%) | 2 (1.1%) | 0 (0%) | 0 (0%) | 0 (0%) | 1 (0.6%) |
| Ectoparasite | 136 (77.7%) | 32 (18.3%) | 3 (1.7%) | 2 (1.1%) | 0 (0%) | 0 (0%) | 0 (0%) | 2 (1.1%) |
| Mixed | 134 (77.9%) | 32 (18.6%) | 3 (1.7%) | 1 (0.6%) | 0 (0%) | 0 (0%) | 1 (0.6%) | 1 (0.6%) |
| Control | 128 (78.0%) | 28 (17.1%) | 5 (3.0%) | 1 (0.6%) | 0 (0%) | 0 (0%) | 0 (0%) | 2 (1.2%) |
|  |  |  |  |  |  |  |  |  |
| **Ethnicity** | | | | | | | | |
| **Treatment** | White | Black/African American | Native American | Asian | Other |  |  |  |
| Pathogens | 133 (77.3%) | 17 (9.9) | 0 (0%) | 15 (8.7%) | 7 (4.1%) |  |  |  |
| Ectoparasite | 137 (783%) | 12 (6.9%) | 2 (1.1%) | 12 (6.9%) | 12 (6.9%) |  |  |  |
| Mixed | 141 (79.7) | 16 (9.0%) | 2 (1.1%) | 10 (5.6%) | 8 (4.5%) |  |  |  |
| Control | 130 (79.3%) | 13 (7.9%) | 1 (0.6%) | 14 (8.5%) | 6 (3.7%) |  |  |  |
| *Categories from the Kinsey scale where 1 = exclusively heterosexual; 2 = predominantly heterosexual but incidentally homosexual; 3 = predominantly heterosexual but more than incidentally homosexual; 4 = Equally heterosexual and homosexual; 5 = homosexual but more than incidentally heterosexual; 6 = homosexual but incidentally heterosexual; 7 = exclusively homosexual; 8 = asexual. | | | | | | | | |
