# Supplementary material for: Microbes and masculinity: Does exposure to pathogenic cues alter women’s preferences for male facial masculinity and beardedness?
Source: PLoS One. 2017 Jun 8;12(6):e0178206. doi: 10.1371/journal.pone.0178206 (PMC5464545; doi:10.1371/journal.pone.0178206)
Supplement: S2 Table — (DOCX) [file pone.0178206.s003.docx]

| **Table S2**. Cronbach’s alphas representing the inter-rater reliability for the 5 stimulus images within each stimulus category for ratings of attractiveness ratings. | | | | | |
| --- | --- | --- | --- | --- | --- |
|  | N | Full beard low masculinity | Full beard high masculinity | Clean-shaven low masculinity | Clean-shaven high masculinity |
| Attractiveness ratings | 688 | 0.92 | 0.91 | 0.91 | 0.90 |
