# Supplementary material for: Microbes and masculinity: Does exposure to pathogenic cues alter women’s preferences for male facial masculinity and beardedness?
Source: PLoS One. 2017 Jun 8;12(6):e0178206. doi: 10.1371/journal.pone.0178206 (PMC5464545; doi:10.1371/journal.pone.0178206)
Supplement: S3 Table — (DOCX) [file pone.0178206.s004.docx]

| **Table S3**. The variance components (random effects) for the models predicting attractiveness ratings when only including pre-manipulation trials. | | | |
| --- | --- | --- | --- |
|  | Empty Model | Predicted Model |  |
| Participant ID |  |  |  |
| Intercept | 253.60 | 189.77 |  |
| Moral Disgust |  | 25.02 |  |
| Sexual Disgust |  | 14.37 |  |
| Pathogen Disgust |  | 20.09 |  |
| Residual | 244.50 | 220.50 |  |
