# Supplementary material for: Microbes and masculinity: Does exposure to pathogenic cues alter women’s preferences for male facial masculinity and beardedness?
Source: PLoS One. 2017 Jun 8;12(6):e0178206. doi: 10.1371/journal.pone.0178206 (PMC5464545; doi:10.1371/journal.pone.0178206)
Supplement: S4 Table — (DOCX) [file pone.0178206.s005.docx]

| **Table S4**. The fixed effects coefficients (and standard errors) and associated *95*% confidence intervals for the influence of moral disgust, sexual disgust, pathogen disgust, as well as sexual dimorphism and beardedness on attractiveness ratings only including pre-manipulation trials. | | |
| --- | --- | --- |
|  | γ (SE) | 95% CI |
| Intercept | 40.45 (.60) | 39.25, 41.64* |
| Moral Disgust | 1.35 (.65) | .06, 2.64* |
| Sexual Disgust | -.88 (.71) | -2.26, .50 |
| Pathogen Disgust | -2.37 (.72) | -3.84, -.94 * |
|  |  |  |
| Preference for Beardedness | 9.11 (.25) | 8.62, 9.61* |
| Moral Disgust | .04 (.27) | -.49, .57 |
| Sexual Disgust | -1.87 (.27) | -2.43, -1.31* |
| Pathogen Disgust | .75 (.29) | .18, 1.32* |
|  |  |  |
| Preference for masculinity | 2.30 (.25) | 1.81, 2.80* |
| Moral Disgust | .65 (.27) | .13, 1.18* |
| Sexual Disgust | .02 (.29) | -.54, .58 |
| Pathogen Disgust | -.46 (.29) | -1.02, .12 |

* 95% confidence interval does not contain 0, indicating statistical significance.
