# Supplementary material for: Microbes and masculinity: Does exposure to pathogenic cues alter women’s preferences for male facial masculinity and beardedness?
Source: PLoS One. 2017 Jun 8;12(6):e0178206. doi: 10.1371/journal.pone.0178206 (PMC5464545; doi:10.1371/journal.pone.0178206)
Supplement: S5 Table — (DOCX) [file pone.0178206.s006.docx]

| **Table S5.** Repeated-measures ANOVA testing the effect of beardedness (clean-shaven, full beard), masculinity (+50%, -50%) and pathogen treatment (ectoparasites, pathogens, mixed, and control) on the differential between women’s post-treatment and pre-treatment attractiveness ratings of male faces. | | | | | |
| --- | --- | --- | --- | --- | --- |
|  | d.f._n_ | d.f._d_ | *F* | *P* | *η_p_^2^* |
| Facial hair | 1 | 684 | 26.29 | <0.001 | 0.037 |
| Facial masculinity | 1 | 684 | 0.10 | <0.754 | <0.001 |
| Treatment | 3 | 684 | 2.10 | 0.099 | 0.009 |
| Facial hair x facial masculinity | 1 | 684 | 0.05 | 0.817 | 0.001 |
| Facial hair x treatment | 3 | 684 | 1.20 | 0.310 | 0.005 |
| Facial masculinity x treatment | 3 | 684 | 0.19 | 0.905 | 0.001 |
| Facial hair x facial masculinity x treatment | 3 | 684 | 0.67 | 0.571 | 0.003 |
