# Supplementary material for: Microbes and masculinity: Does exposure to pathogenic cues alter women’s preferences for male facial masculinity and beardedness?
Source: PLoS One. 2017 Jun 8;12(6):e0178206. doi: 10.1371/journal.pone.0178206 (PMC5464545; doi:10.1371/journal.pone.0178206)
Supplement: S6 Table — (DOCX) [file pone.0178206.s007.docx]

| **Table S6**. Repeated-measures ANOVA, with the beard (clean-shaven, full beard), masculinity (+50%, -50%) and time (pre, post) as within-subjects factors and pathogen treatment (ectoparasites, pathogens, mixed, and control) and relationship status (in a relationship, single) as between-subjects factors. | | | | | |
| --- | --- | --- | --- | --- | --- |
|  | d.f._n_ | d.f._d_ | *F* | *P* | *η_p_^2^* |
| Facial hair | 1 | 680 | 233.24 | <0.001 | 0.255 |
| Facial masculinity | 1 | 680 | 51.96 | <0.001 | 0.071 |
| Time | 1 | 680 | 1.56 | 0.212 | 0.002 |
| Treatment | 3 | 680 | 2.10 | 0.099 | 0.009 |
| Relationship status | 1 | 680 | 0.36 | 0.550 | 0.001 |
| Relationship status x treatment | 3 | 680 | 0.54 | 0.653 | 0.002 |
| Relationship status x time | 1 | 680 | 0.63 | 0.428 | 0.001 |
| Facial hair x facial masculinity | 1 | 680 | 11.47 | <0.001 | 0.017 |
| Facial hair x treatment | 3 | 680 | 1.31 | 0.271 | 0.006 |
| Facial hair x time | 1 | 680 | 19.09 | <0.001 | 0.027 |
| Facial hair x relationship status | 1 | 680 | 0.05 | 0.833 | <0.001 |
| Facial masculinity x treatment | 3 | 680 | 1.14 | 0.334 | 0.005 |
| Facial masculinity x time | 1 | 680 | 0.10 | 0.750 | <0.001 |
| Facial masculinity x relationship status | 1 | 680 | 9.60 | 0.002 | 0.014 |
| Time x treatment | 3 | 680 | 2.80 | 0.039 | 0.012 |
| Facial hair x facial masculinity x treatment | 3 | 680 | 0.93 | 0.424 | 0.004 |
| Facial hair x facial masculinity x time | 1 | 680 | 0.15 | 0.698 | <0.001 |
| Facial hair x time x treatment | 3 | 680 | 0.07 | 0.977 | <0.001 |
| Facial hair x time x relationship status | 1 | 680 | 0.08 | 0.785 | <0.001 |
| Facial hair x facial masculinity x relationship status | 1 | 680 | 0.09 | 0.759 | <0.001 |
| Facial hair x treatment x relationship status | 3 | 680 | 1.81 | 0.144 | 0.008 |
| Facial masculinity x time x treatment | 3 | 680 | 0.60 | 0.613 | 0.003 |
| Facial masculinity x time x relationship status | 1 | 680 | 0.03 | 0.853 | <0.001 |
| Facial masculinity x treatment x relationship status | 3 | 680 | 0.58 | 0.630 | 0.003 |
| Relationship status x time x treatment | 3 | 680 | 1.13 | 0.334 | 0.005 |
| Facial hair x facial masculinity x time x treatment | 3 | 680 | 0.97 | 0.407 | 0.004 |
| Facial hair x facial masculinity x treatment x relationship status | 3 | 680 | 1.193 | 0.312 | 0.005 |
| Facial hair x time x treatment x relationship status | 3 | 680 | 1.35 | 0.256 | 0.006 |
| Facial masculinity x time x treatment x relationship status | 3 | 680 | 0.95 | 0.416 | 0.004 |
| Facial hair x facial masculinity x time x relationship status | 1 | 680 | 0.99 | 0.320 | 0.001 |
| Facial hair x facial masculinity x treatment x time x relationship status | 3 | 680 | 0.99 | 0.398 | 0.004 |
