# Supplementary material for: Microbes and masculinity: Does exposure to pathogenic cues alter women’s preferences for male facial masculinity and beardedness?
Source: PLoS One. 2017 Jun 8;12(6):e0178206. doi: 10.1371/journal.pone.0178206 (PMC5464545; doi:10.1371/journal.pone.0178206)
Supplement: S7 Table — (DOCX) [file pone.0178206.s008.docx]

| **Table S7**. The variance components (random effects) for the models predicting attractiveness ratings for Analysis 2. | | |
| --- | --- | --- |
|  | Empty Model | Predicted Model |
| Participant ID |  |  |
| Intercept | 255.90 | 188.68 |
| Moral Disgust |  | 29.65 |
| Sexual Disgust |  | 14.45 |
| Pathogen Disgust |  | 21.55 |
| Residual | 251.60 | 225.00 |
